# Free-Breathing, Non-Gated Heart-To-Brain CTA in Acute Ischemic Stroke: A Feasibility Study on Dual-Source CT

**DOI:** 10.3389/fneur.2022.616964

**Published:** 2022-02-22

**Authors:** Jiabin Liu, Chen Wang, Qing Li, Xianggong Duan, Xiaolian Zhu, Jiahong Wang, Xiangying Du, Jie Lu, Kuncheng Li

**Affiliations:** ^1^Department of Radiology, Xuanwu Hospital, Capital Medical University, Beijing, China; ^2^Beijing Key Laboratory of Magnetic Resonance Imaging and Brain Informatics, Beijing, China

**Keywords:** computed tomography, dual-source CT, CT angiography, stroke, cardiac imaging techniques

## Abstract

**Purpose:**

To validate the feasibility of free-breathing, non-gated, high-pitch heart-to-brain computed tomography arteriography (CTA) in acute ischemic stroke and the capability of non-gated heart-to-brain CTA in showing cardiac anatomy.

**Materials and Methods:**

The study protocol was approved by the institutional medical ethics review board. Free-breathing, non-gated, high-pitch heart-to-brain CTA was performed on patients with acute ischemic stroke referred for multimodal CT using a third-generation dual-source CT. Patients scheduled for ECG-triggered heart-to-brain CTA served as controls. Quantitative and/or qualitative image quality of the four cardiac chambers, left atrial appendage, interventricular and interatrial septa, carotid arteries, and coronary arteries were evaluated and compared between the two groups.

**Results:**

Free-breathing, non-gated, high-pitch heart-to-brain CTA was performed on 30 patients with acute ischemic stroke, whereas the control group included 31 cases. There is no significant difference in the image quality of CTAs between the two groups at cardiac chambers and carotid arteries. The image quality of coronary arteries also showed no significant difference between the two groups. The mean dose length products of CTA in the two groups were 129.1 ± 30.5 mGy cm and 121.6 ± 30.3 mGy cm, respectively. Cardiac abnormality can be shown in patients with acute ischemic stroke.

**Conclusion:**

It is feasible to use free-breathing, non-gated, high-pitch heart-to-brain CTA with dual-source CT in acute ischemic stroke for cardiac etiology screening.

## Introduction

Ischemic stroke has various etiologies, including large-artery atherosclerosis, cardiogenic emboli, small-vessel occlusion, and other determined or undetermined etiology (1). Because of increased concern about atherosclerosis and hyperlipidemia, the primary prevention of ischemic stroke has been improved through recent years. Although the overall incidence rate of ischemic stroke decreased, the incidence of cardiogenic stroke has increased (2). In addition, patients with cardiogenic stroke showed more severe presentations and worse prognoses (3). Therefore, the identification of cardiogenic stroke should be emphasized in the management of patients with acute ischemic stroke, especially for the possible etiology of cardiogenic stroke. In clinical practice, transesophageal echocardiography (TEE) is more effective than transthoracic echocardiography (TTE) in patients suspected of intracardiac thrombus (4, 5). However, as a semi-invasive procedure, TEE needs adequate preparation, which makes it inappropriate for patients with acute ischemic stroke when emergent management may be needed (6).

Computed tomography perfusion (CTP) combining computed tomography arteriography (CTA) of cerebral and carotid arteries has been used as the second-line imaging to non-enhanced brain CT for patients with ischemic stroke, mainly for large vessel diseases (7, 8). Recently, the combined CTA from heart-to-brain using high-pitch spiral acquisition with dual-source CT was introduced to evaluate the overall atherosclerotic burden, which may be useful for the prediction of prognosis for patients after ischemic stroke or acute cardiac events (9). With appropriate equipment and protocol, it may also be performed in emergency settings, adding information of morphological changes of the heart for differential diagnosis of cardiogenic stroke. We intended to validate the feasibility of free-breathing, non-gated emergent high-pitch heart-to-brain CTA after the brain perfusion scan using a third-generation dual-source CT scanner and the capability of non-gated heart-to-brain CTA in showing cardiac anatomy.

## Materials and Methods

### Study Population

The study protocol was approved by the institutional medical ethics review board. Patients with acute ischemic stroke who were referred for multimodal CT from March 2018 to December 2018 were recruited consecutively, and informed consent was obtained from each patient or his/her representative. Another group of patients with ischemic stroke who were scheduled for heart-to-brain CTA in this period were used as controls. Standard exclusion criteria for contrast-enhanced CTA included: previous allergic reaction to iodinated contrast, renal disease with serum creatinine level higher than 1.5 mg/ml, and detected cerebral hemorrhage by a non-enhanced scan.

### CT Protocol

The CT scans were performed with a third-generation dual-source CT scanner (SOMATOM Force, Siemens Healthcare, Forchheim, Germany).

Combined CTP and non-gated high-pitch heart-to-brain CTA protocol consisted of four steps. (1) Anteroposterior and lateral localizing topogram, (2) Non-contrast brain CT, (3) Perfusion CT. The acquisition parameters were as follows: 2 × 96 × 0.6 mm collimation, 80 kV, 50 mA, 0.25 s gantry rotation time, 114 mm coverage, and 5 mm slice. The dual-phase contrast injection protocol consists of 40 ml of iodinated contrast (Ultravist 370 mgI/ml) injected through the right or left (preferably the right) antecubital vein and followed by saline flush of 40 ml. The injection rate was 5 ml/s for both the contrast and saline. (4) Free-breathing, non-gated, and high-pitch heart-to-brain CTA. The acquisition parameters were as follows: collimation 2 × 96 × 0.6 mm, 90 kV tube voltage, reference tube current-time product 90 mA, 4D dose modulation (Care Dose 4D, Siemens Healthcare), and a pitch of 3.2. The rotation time was 0.25 s. The acquisitions were obtained in the caudocranial direction from the diaphragm to the vertex without breath-holding, and no ECG wire was connected. The injection protocol consisted of 60 ml of iodinated contrast, followed by 40 ml of saline. The injection rate was 5 ml/s for both the contrast and the saline. Scan timing was achieved with bolus-tracking technique with a monitoring region of interest (ROI) placed in the ascending aorta at the level of the left main coronary artery (threshold 100 HU). The CTA data acquisition was initiated with a delay of 6 s.

The scheduled heart-to-brain CTA protocol consisted of topogram, non-contrast heart-to-brain CT, and ECG-triggered high-pitch heart-to-rain CTA under breath-holding after inspiration. For patients with a heart rate under 70 bpm, the trigger phase was set at a 60% RR interval and for those with a heart rate higher than 70 bpm, the trigger phase was set at a 20% RR interval (10, 11). The contrast protocol and the other scan parameters were the same as the non-gated CTA.

### Image Preparation

Images of 0.6-mm slice thickness were reconstructed from raw data using Advanced Modeled Iterative Reconstruction (ADMIRE, strength 3) and transferred to the workstation (MMWP, Siemens Healthcare). A medium-smooth tissue convolution kernel (Bv36) was used. Curved planar reformation (CPR), maximum intensity projection (MIP), and multiplanar reformation (MPR) were used for further evaluation of the carotid, cerebrovascular, coronary arteries, and cardiac chambers.

#### Image Quality Evaluation

Qualitative and/or quantitative evaluation of image quality was performed for carotid CTA, coronary CTA, and cardiac morphology. Parameters for quantitative image quality evaluation included absolute contrast enhancement and contrast-to-noise ratio.

#### Carotid Arteries, Jugular Veins, and Aorta

The mean attenuation of vessels was measured by placing a spherical ROI (200 mm^2^ for aorta and 2–3 mm^2^ for the other locations) within each contrast-enhanced lumen. ROIs were also placed in the connective tissue surrounding each vessel lumen.

Image noise was defined as the SD of the mean attenuation of an ROI in the aortic root at the level of the left coronary sinus. CNR was then calculated by using the following equation (12):

CNR = (CT Attenuation Lumen–CT Attenuation Connective Tissue)/Image Noise.

Quantitative measurements were performed at the following locations: bilateral internal carotid artery (ICA) (3 cm above carotid bifurcation), bilateral common carotid artery (CCA) (3 cm below carotid bifurcation), bilateral vertebral artery, ascending aorta, descending aorta, superior vena cava, and bilateral internal jugular veins. The measurements of superior vena cava, ascending aorta, and descending aorta were performed at the level of the right pulmonary artery. ROIs in the internal jugular veins were placed at the level of the superior margin of the hyoid bone (13).

#### Coronary Arteries

Objective evaluation of coronary arteries was performed on the 15 segments according to the standards of the American Heart Association (AHA) (14). All the measurements of coronary segment enhancement were performed by placing an ROI of 2–3 mm^2^ in the lumen avoiding calcifications and plaques. Background attenuation was measured by placing an ROI of 2–3 mm^2^ in the adjacent tissue. Then CNR of each segment was calculated.

Subjective evaluation was performed by two independent radiologists with more than 5 years of experience in cardiovascular imaging, without knowledge of the scan protocol.

All the images were transferred to the workstation and evaluated in random order. Patient information and scan protocol were concealed. Discrepancies in image assessment by the two radiologists, if any, were discussed and resolved by consensus. A 15-segment classification criterion from AHA was used to analyze the coronary artery segment by segment. The image quality was analyzed with a 4-point method as follows: 1 point, severe image artifacts with a poor demonstration of the coronary artery, and the boundary of the blood vessel is vague; 2 points, mid-level artifacts in images with relatively good contrast between the coronary artery and surrounding tissues; 3 points, light level of image artifacts, good contrast between the coronary artery and surrounding tissues with a clear demonstration of the blood vessel; and 4 points, good demonstration of coronary artery, clear and sharp edge is also shown.

#### Cardiac Structure

An objective evaluation was performed on each cardiac chamber and left atrial appendage (LAA) by placing an ROI of up to 100 mm^2^ in the lumens. ROIs of about 10 mm^2^ were placed in the septa to measure the background attenuation. CNRs were then calculated by using the equation mentioned above.

The image quality at LAA and septum was also evaluated subjectively with a 3-point scale: 1 point, poor contrast or severe motion artifact that could not be used to analyze; 2 points, mid-level artifacts and relatively good contrast; and 3 points, good contrast and without motion artifacts.

#### Radiation Dose

Volume CT dose index (CTDI_VOL_) and dose-length product (DLP) of the CTAs were recorded.

### Statistical Analysis

All the statistical analyses were performed using commercially available software Statistic Package for Social Science (SPSS, release 20.0, SPSS, USA). Continuous variables were expressed as mean ± SD and evaluated with an independent sample *t*-test. Categorical variables were expressed as frequencies or percentages and compared with the chi-square test. A *p*-value below 0.05 was considered statistically significant.

## Results

### Patient Characteristics

The emergent non-gated CTA group included 30 patients (26 men, 4 women). About 31 patients (29 men, 2 women) were recruited in the control group. There was no statistically significant difference between the two groups in age, BMI, and heart rate (*p* > 0.05). Among the 30 patients with acute ischemic stroke, two cases of LA/LAA thrombi, one case of patent foramen ovale, and one case of hemostasis in LAA were found (Table 1).

**Table 1:** Patient characteristics.

	**Male/Female**	**Age (yrs)**	**Body weight (kg)**	**BMI**	**Heart rate (bpm)**
Non-gated group	26/4 (*n* = 30)	61.5 ± 8.6	75.3 ± 12.6	26.8 ± 4.	75.1 ± 8.6
ECG-triggered group	29/2 (*n* = 31)	61.1 ± 7.2	72.6 ± 9.3	25.0 ± 2.7	74.2 ± 7.9

*BMI, body mass index; bpm, beat per minute*.

### Image Quality

There was no significant difference in the quantitative image quality between the two groups in bilateral ICA, bilateral CCA, bilateral vertebral arteries, ascending aorta, and descending aorta. The mean attenuation values in all the arteries were more than 450 HU, all the mean CNR values were more than 20, and the mean attenuation values in veins were <200 HU (Tables 2, 3).

**Table 2:** Quantitative analysis of image quality (vessel enhancement, Hu).

	**Non-gated**	**ECG-triggered**	***p*-value**
	**group**	**group**	
L-CCA	553.7 ± 98.7	576.9 ± 86.6	0.334
R-CCA	554.2 ± 105.8	591.1 ± 84.5	0.137
L-ICA	559.7 ± 96.6	584.8 ± 82	0.297
R-ICA	563.5 ± 102.3	586.3 ± 94.6	0.390
L-VA	488.3 ± 81.9	510.9 ± 127.1	0.422
R-VA	479.1 ± 103.3	526.5 ± 103.9	0.079
AAO	541.5 ± 81.9	566.9 ± 77.3	0.216
DAO	537.6 ± 87.5	559.4 ± 80.4	0.315
SVC	205.3 ± 70.4	163.5 ± 43	0.007
L-IJV	151.6 ± 70.9	173.5 ± 86.8	0.285
R-IJV	153.4 ± 63.3	184.3 ± 89.2	0.125

*CCA, common carotid artery; ICA, internal carotid artery; VA, vertebral artery; CNR, contrast to noise ratio; AAO, Ascending aorta; DAO, Descending aorta; SVC, Superior vena cava; IJV, internal jugular veins*.

**Table 3:** Quantitative analysis of image quality (CNR).

	**Non-gated**	**ECG-triggered**	***p*-value**
	**group**	**group**	
L-CCA	26.5 ± 7.1	26 ± 6.9	0.768
R-CCA	26 ± 7.3	26 ± 6.7	0.981
L-ICA	25.6 ± 8	26.1 ± 6.8	0.795
R-ICA	25 ± 8	25.5 ± 9	0.839
L-VA	22.7 ± 7.1	21 ± 7.6	0.382
R-VA	21.7 ± 7.2	22.5 ± 6.8	0.655
AAO	26 ± 7.6	27.3 ± 6.1	0.451
DAO	23.8 ± 7.8	25 ± 5.9	0.476
SVC	12.2 ± 4.9	9.3 ± 2.6	0.005
L-IJV	11.8 ± 6.5	10.2 ± 4.2	0.249
R-IJV	11.9 ± 5.5	11 ± 5.1	0.519

*CCA, common carotid artery; ICA, internal carotid artery; VA, vertebral artery; CNR, contrast to noise ratio; AAO, Ascending aorta; DAO, Descending aorta; SVC, Superior vena cava; IJV, internal jugular veins*.

**Figure 1:**
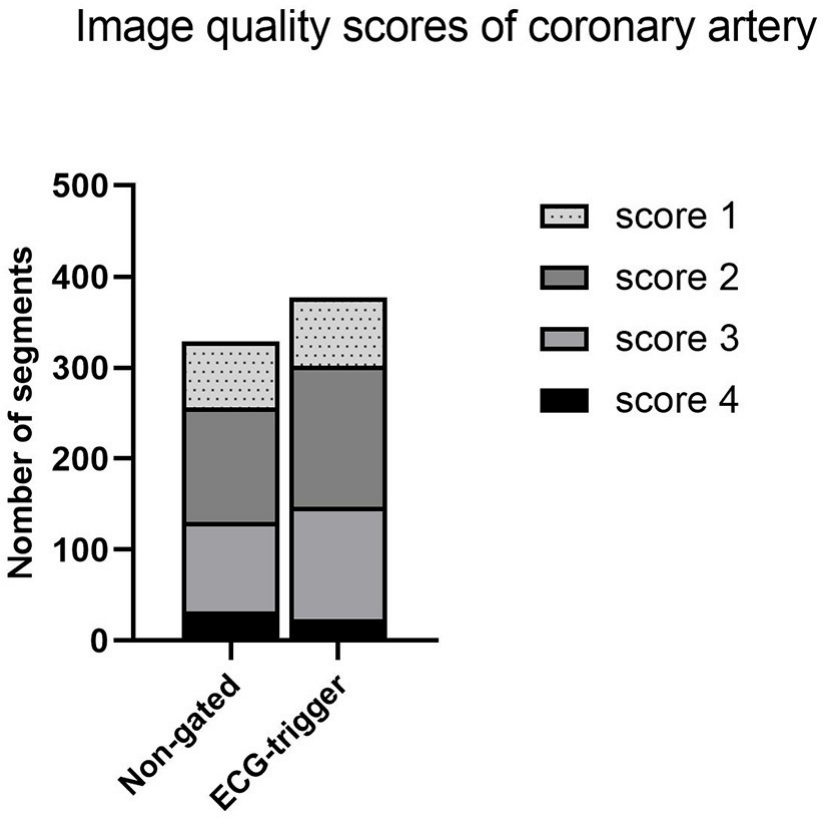
Image quality scores of the coronary artery.

As for coronary artery image quality, a total of 706 coronary artery segments were evaluated (non-gated 329 and ECG-gated 377), and there was no significant difference between the two groups in the mean attenuation and the mean CNR values (Figure 1).

In the non-gated group, the mean attenuation values of cardiac chambers were as follows: LA 429.07 ± 96.4, LV 444.1 ± 87.4, RA 174.2 ± 67.0, RV 188.3 ± 70.4, and LAA 463.4 ± 127.0. In the ECG-gated group, the mean attenuation values were as follows: LA 425.8 ± 112.1, LV 483.4 ± 80.4, RA 129.3 ± 31.2, RV 131.7 ± 39.3, and LAA 490.8 ± 84.2. There was no significant difference between the two groups in the mean attenuation, LA and LAA, whereas there was a statistical difference at RV and RA (Table 4).

**Table 4:** Quantitative analysis of each chamber and LAA (mean attenuation, Hu).

	**LA**	**LV**	**RA**	**RV**	**LAA**
Non-gated group	429.1 ± 96.4	444.1 ± 87.4	174.2 ± 67.0	188.3 ± 70.4	463.4 ± 127.0
ECG-triggered group	425.8 ± 112.1	483.4 ± 80.4	129.3 ± 31.2	131.7 ± 39.3	490.8 ± 84.2
*p*-value	0.905	0.072	0.002	0.000	0.320

*LA, left atrium; LV, left ventricle; RA, right atrium; RV, right ventricle; LAA, left atrial appendage*.

**Figure 2:**
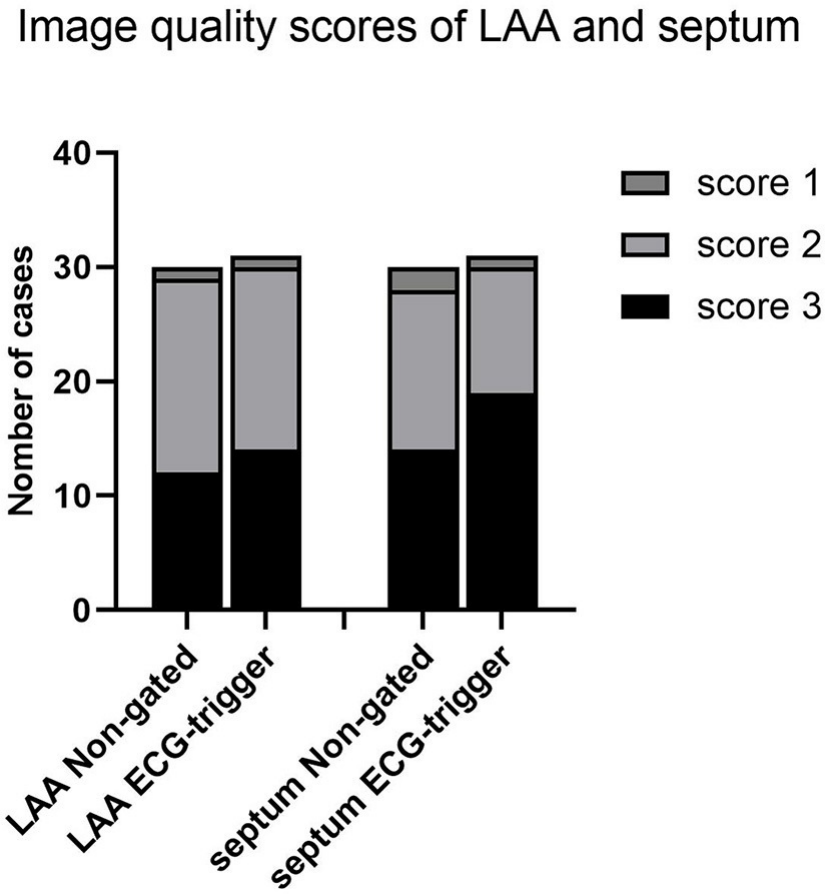
Image quality scores of the left atrial appendage (LAA) and septum.

There was no significant difference between the two groups in qualitative evaluation of motion artifact at LAA and interatrial septum (Figure 2).

#### Radiation Dose

The mean CTDI_vol_ and DLP of CTA scan in the non-gated group were 2.6 ± 0.6 and 129.1 ± 30.5 mGy cm, respectively. The mean CTDI_vol_ and DLP of CTA scan in the ECG-gated group were 2.4 ± 0.6 and 121.6 ± 30.3 mGy cm, respectively. There was no significant difference (*p* = 0.152 and 0.344, respectively).

## Discussion

Because of the elevated incidence of cardiogenic stroke, the need for evaluation of possible etiology of cardiogenic emboli has increased. Therefore, TEE has been increasingly used for stroke patient evaluation (15). However, a possible cardiac etiology detection may be needed at the first presence of patients with acute stroke at the Emergency Department, which makes it difficult for conducting TEE evaluation (6). Multimodal CT including brain CTP and CTA has been preferred for the evaluation of acute ischemic stroke (16–18). If the heart can be covered in the CTA scan, it would be possible to perform a cardiac evaluation in emergency settings. Tognolini et al. has tried to perform combined cardiac and cerebral CTA with 64-slice CT scanner and suggested that the protocol had the potential for comprehensive and time-efficient imaging of both carotid and coronary arteries (19). However, the scan protocol with 64-slice CT might prolong the total examination time, which may not be permitted for patients with emergent stroke. To overcome these limitations, Molina-Fuentes et al. designed an ECG-gated CT protocol using a wider detector CT (256-row) for the assessment of thoracic cardiovascular thrombi in addition to the brain perfusion and cerebral arteries in acute ischemic stroke (20). No significantly elevated time-consuming or radiation exposure was found with the extended protocol, whereas better detection of some thrombi over TTE or TEE might be achieved, providing a promising solution for the emergent cardio-stroke imaging with the wide detector CT.

Studies have also suggested that a dual-source CT can be used to speed up CTA scans with specific protocols. Sun et al. introduced an ECG-triggered high-pitch spiral scan CTA to cover the range from the heart to the brain using the second-generation dual-source CT scanner and proved the feasibility and image quality in non-emergent patients (9). However, an ECG-incorporated scan with breath-holding may need more preparation time, especially for patient training, which may be not possible in emergent patients, especially for those with unconsciousness or unstable heart rate. In addition, an unstable heart rate that frequently occurred in emergent patients may cause incorrect timing of ECG-triggered scans and deteriorate the image quality of cardiac structures, especially the coronary arteries. Non-gated high-pitch (pitch 2.8/3.0) protocol has been used to minimize motion artifact in CTAs for acute pulmonary embolism or aortic dissection (21, 22). Choi et al. also found that image quality may not be hampered at the proximal and mid-coronary segments in non-gated scans (23). As for breathing, studies have proved that breath may not influence the image quality of coronary CTA when it was performed in one heart beat (24). Therefore, we intended to incorporate heart-to-brain CTA without breath-holding and ECG gating into the multimodal CT for acute ischemic stroke, saving time for preparation, with comparable scan time to the existing scan protocol.

Another concern for increased scan range would be the radiation dose. Former studies have proved that the radiation dose of high-pitch heart-to-brain CTA is pretty low (25). In comparison with the radiation dose for the brain CTP and the other CTA protocols for carotid and brain CTA or multimodal CT in acute ischemic stroke, the radiation dose is significantly lower, given the fact that incorporating this CTA protocol did not increase the total radiation dose.

**Figure 3:**
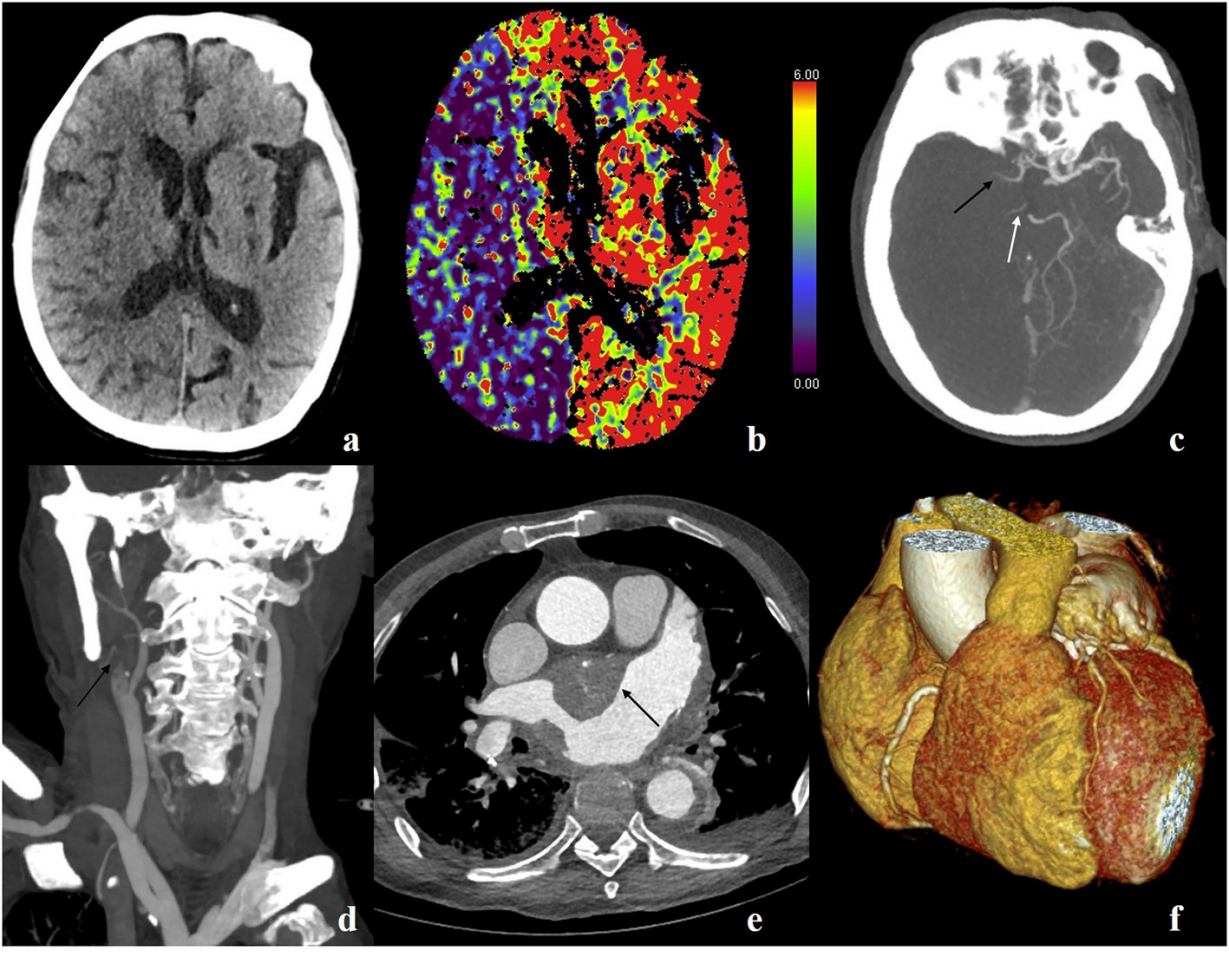
A 73-year-old male, suffered sudden generalized convulsion and unconsciousness for 4 h, with a history of hypertension, rheumatic heart disease, and atrial fibrillation. **(a)** Non-contrast scan shows decreased attenuation in the right hemisphere and blurred gray–white matter boundary. **(b)** Cerebral blood volume map shows decreased blood volume in the right hemisphere. **(c)** Maximum intensity projection (MIP) image shows the occluded right middle cerebral artery (black arrow) and posterior cerebral artery (white arrow). **(d)** MIP image shows occluded right internal carotid artery (black arrow). **(e)** Axial image shows a massive thrombus in the left atrium (black arrow). **(f)** VR image of the heart and coronary arteries.

Many studies have proved that a large proportion of cardiogenic and cryptogenic strokes may be related to the underlying abnormality of the left atrium, especially the LAA and interatrial septum. These abnormalities include thrombus in the LAA, hemostasis in the LAA, patent foramen ovale, interatrial septal aneurysm, etc. The detection of these abnormalities depends on adequate illustration of cardiac chambers and the septa, especially the structures related to the left atrium. As the in-plane temporal resolution of the high-pitch scan is very high (66 ms) for the third-generation dual-source CT scanner, although the image quality of coronary arteries might be affected, the image quality of larger structures as cardiac chambers might be sustained. In our study, the image quality of LA, LAA, and interatrial septum is acceptable, with no difference between ECG-triggered and not-gated scans. In addition, even the study was not designed specifically for suspected cardiogenic stroke, and abnormalities of cardiac chambers were detected in some cases (Figure 3).

Besides the cardiac chambers, the image quality of carotid vessels was also adequate. This proved that no deterioration of the evaluation of carotid and cerebral vessels occurred when incorporating the cardiac scan into the existing CTA protocol. Interestingly, the image quality of coronary arteries was also comparable to the ECG-triggered high-pitch CTA. Based on the theoretical basis of the high-pitch scan, although non-gated scans were purely randomly timed, it has a good chance to meet at least part of the relative static phase. The high in-plane temporal resolution of the high-pitch scan may be another reason.

As the first study to evaluate the feasibility to incorporate a high-pitch heart-to-brain CTA scan into the multimodal CT protocol for acute ischemic stroke, limitations may still exist. As the sample size is small and not dedicated to suspected cardiogenic stroke, only 4 cases with positive findings were found. The profit of this protocol needs to be further evaluated. Second, as the CTA scan was just a single cardiac phase and single contrast phase scan, cardiac function data which may be valuable for therapeutic strategy and prognosis prediction cannot be obtained, and the differentiation between LAA thrombus and hemostasis may be difficult. Third, at present, the high-pitch protocol is only available on the second-and third-generation dual-source CT scanner, the possibility of heart-to-brain CTA in the emergent department needs to be verified while these scanners are not on site.

In summary, we introduced a free-breathing, non-gated, and high-pitch heart-to-brain CTA protocol incorporated in the multimodal CT of acute ischemic stroke and validated the feasibility and image quality, without an increase of radiation dose. Prolonged CTA range can provide more information and be profitable for patients with acute ischemic stroke, especially for those suspected of cardiogenic stroke or embolic stroke of undetermined source.

## Data Availability Statement

The datasets presented in this study can be found in online repositories. The names of the repository/repositories and accession number(s) can be found in the article/Supplementary Material.

## Ethics Statement

The studies involving human participants were reviewed and approved by the Institutional Medical Ethics Review Board, Xuanwu Hospital. The patients/participants provided their written informed consent to participate in this study.

## Author Contributions

XDu, KLi, and JLu: conceived and designed the experiments. JLiu, XZhu, and XDuan: performed the experiments. JLiu, CW, JW, and QLi: analyzed the data. JLiu: wrote the manuscript. All authors contributed to the article and approved the submitted version.

## Funding

This study was supported by the grant from the Beijing Municipal Administration of Hospitals Clinical Medicine Development of Special Funding (ZYLX201609), the Beijing Municipal Science and Technology Commission (Z161100000516087), and the Beijing Municipal Administration of Hospitals' Ascent Plan (DFL20180802).

## Conflict of Interest

The authors declare that the research was conducted in the absence of any commercial or financial relationships that could be construed as a potential conflict of interest.

## Publisher's Note

All claims expressed in this article are solely those of the authors and do not necessarily represent those of their affiliated organizations, or those of the publisher, the editors and the reviewers. Any product that may be evaluated in this article, or claim that may be made by its manufacturer, is not guaranteed or endorsed by the publisher.
